# Colonic Volvulus Associated with Hirschsprung’s Disease in the Pediatric Age

**DOI:** 10.1007/s00384-025-04994-8

**Published:** 2025-09-15

**Authors:** Hazem Samir Amra, Mostafa M. Elghandour, Mohammed Abdel-Latif

**Affiliations:** 1 Pediatric Surgery Department, Faculty of Medicine, Ain Shams University, Cairo, Egypt; 2 Pediatric Surgery Department, Faculty of Medicine, Helwan University, Cairo, Egypt

**Keywords:** Constipation, Intestinal obstruction, Contrast enema, Coffee bean sign

## Abstract

**Purpose:**

Colonic volvulus (CV) is a twist of part of the colon over its mesentery. Although CV is rare in children, its incidence is unknown. Hirschsprung’s disease (HD) represents a significant risk factor of CV in children, especially when diagnosed late.

**Aim:**

To review the clinical, radiological and management data of children with CV associated with HD.

**Methods:**

Medical records were reviewed from January 2000 to December 2022 looking for children had CV associated with HD.

**Results:**

21 cases (17 males and 4 females) were admitted with CV. Their ages ranged from 8 days to 14 years. Sigmoid volvulus was recorded in 17 (81%) cases, while 4 (19%) cases had cecal volvulus. CV was associated with HD in 9 (42.9%) cases. Their median age was 7 years. Eight cases had sigmoid volvulus associated with short segment HD, while one case had cecal volvulus with long segment HD. CV was the first presentation, before the diagnosis of HD, in 8 cases. The diagnosis of HD was overlooked in 3 cases; 2 cases had an anastomotic leakage after sigmoidectomy, while the third case had recurrence of volvulus after successful nonoperative management.

**Conclusion:**

The diagnosis of CV in children mandates a high index of suspicion. Moreover, HD should be suspected and excluded in every case of CV in children.

## Introduction

Colonic volvulus (CV) is realized as a twist of any part of the colon over its attached mesentery causing bowel obstruction and impairment of the blood supply of the affected segment [1]. It represents the third cause of large bowel obstruction in adults [2]. If not diagnosed and managed in the proper time, it can progress to colonic ischemia, necrosis and finally life-threatening sepsis.

Colonic volvulus is rare in the pediatric age. Its true incidence is unknown. The most affected part is the sigmoid colon followed by the cecum and the transverse colon respectively [3]. Although there is no clear etiology of CV, the presence of redundant hypomobile colon, elongated mesocolon and relatively narrow fixation points may facilitate CV. Hirschsprung’s disease (HD) represents a significant risk factor of CV in the pediatric age, especially when diagnosed in late infancy or afterwards [4].

In the current work we aimed to review the clinical, radiological and management data of pediatric cases with CV associated with HD.

## Patients and methods

The medical records from January 2000 to December 2022 were checked to count pediatric cases who were admitted with CV. The onset of symptoms, presentation, radiological and initial management data were reviewed. Cases with associated HD, which had been eventually diagnosed by radiological and pathological investigations, were identified. Their detailed demographic, clinical, radiological, management and outcome data were considered. Moreover, the total number of newly diagnosed cases with HD were checked during the same period.

## Results

During the study period, 21 cases (24 episodes) were admitted with CV, comprising 17 males and four females. Their ages ranged from 8 days to 14 years. Sigmoid volvulus was recorded in 17 (81%) cases, while 4 (19%) cases had cecal volvulus. Three cases with sigmoid volvulus had a single episode of recurrence.

CV was associated with HD in 9 cases, comprising eight males and one female. Their ages ranged from 8 days to 11 years (median: 7 years, IQR: 8.5–5.5). Eight cases (9 episodes) had sigmoid volvulus associated with short segment HD (one had sigmoid perforation). Additionally, one case had cecal volvulus associated with long segment HD. CV occurred in all these cases before the HD pull through surgery, whereas it was the first presentation, before the diagnosis of HD, in 8 patients. During the same study period, HD was newly diagnosed with 569 patients. The association between CV and HD was found in 42.9% of CV cases, while it was present in 1.6% of HD cases.

CV was detected in 12 (57.1%) cases due to factors unrelated to HD, comprising nine males and three females. Their ages ranged from 8 to 14 years. Nine cases (11 episodes) had sigmoid volvulus, whereas 3 had cecal volvulus. The underlying etiologies were functional constipation according to Rome IV criteria [5] in 5 (23.8%) cases, cerebral palsy (CP) in 4 (19%) cases and other causes, namely congenital malfixation, Down syndrome or neuronal intestinal dysplasia, in 3 (14.3%) cases, respectively.

All cases with CV were presented 2 to 5 days after the start of their symptoms. All cases presented with acute abdominal pain, distension and absolute constipation. Nausea and/or vomiting were present in 16 (76.2%) cases. Peritonitis was diagnosed in one case based on the clinical presentation. A total of 20 cases underwent digital rectal examination. All of them had an empty rectum. There was a history of constipation or delayed meconium passage in 20 (95.2%) cases. Thirteen (61.9%) cases reported previous similar episodes that were relieved either spontaneously or by large-volume enema at home.

**Fig. 1:**
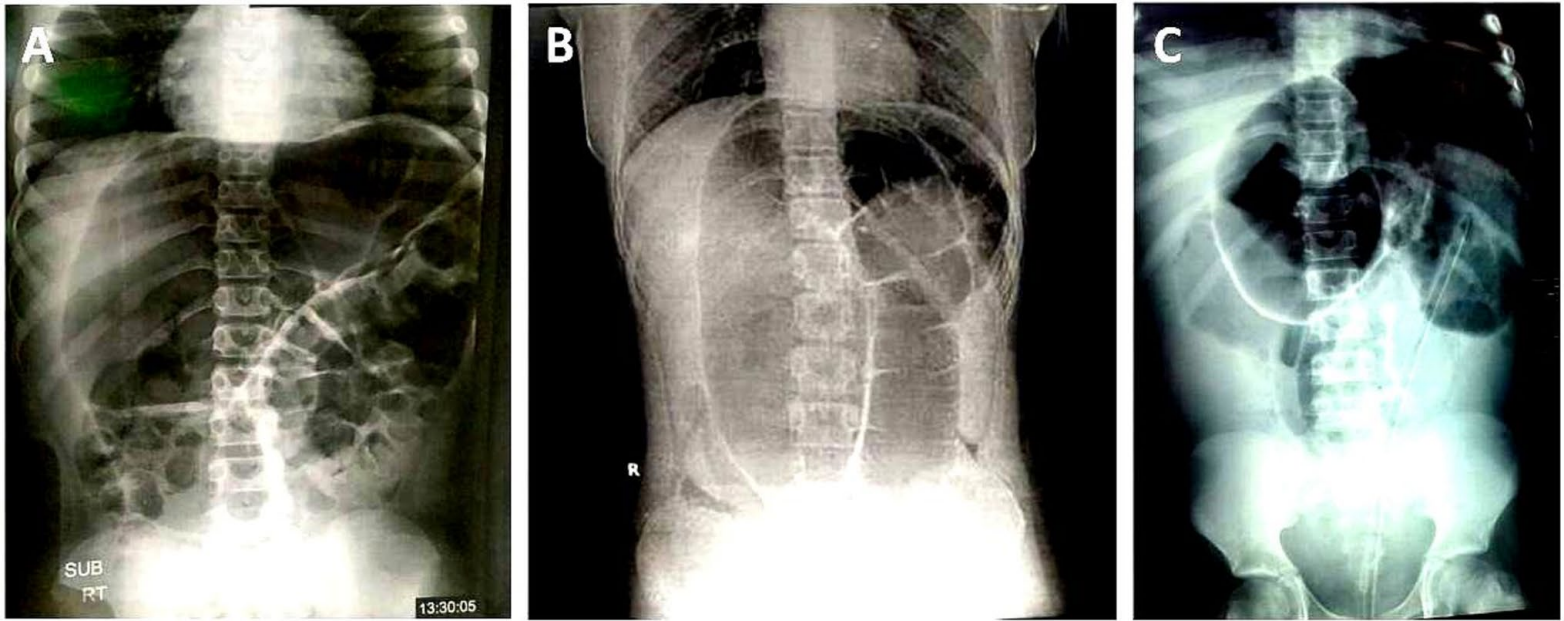
Three different supine abdominal X-rays showing Coffee Bean Sign (massively dilated and gas-filled sigmoid colon which formed inverted U-shaped shadow) and Liver Overlap Sign (the distended sigmoid extends and projects over the liver shadow). **A**) A case of Hirschsprung disease, **B**) A case of chronic constipation, **C**) A case of chronic constipation with rectal tube insertion

**Fig. 2:**
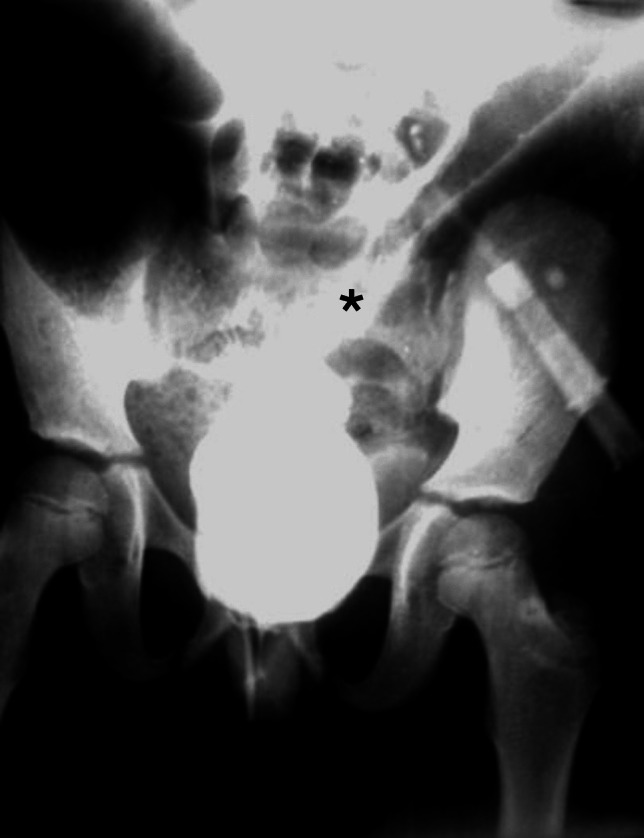
Contrast enema for a case of colonic volvulus secondary to chronic constipation showing Bird’s Beak Sign (*) where the sigmoid colon is gradually narrowed and tapered

Plain abdominal radiographs showed localized massive bowel dilatation in 20 cases. Absent distal aeration was an associated finding in 14 cases, while there were some distal aerations in 6 cases. Free air under the diaphragm was detected with perforated sigmoid volvulus in one case. The coffee bean sign was detected in one case, while it was combined with the liver overlap sign in another 3 cases (Fig. 1). A contrast enema was performed to confirm the diagnosis of 18 cases, revealing bird’s beak deformity in 17 cases (Fig. 2).

Nonoperative management, in the form of fluoroscopic guided contrast enema followed by rectal tube insertion for 24 h, was the initial treatment in cases with sigmoid volvulus without signs of peritonitis or bowel perforation. It was done in 16 cases (19 episodes); 7 cases (8 episodes) had HD, and 9 cases (11 episodes) had constipation due to other causes. Nonoperative management achieved 73.7% global success rate; 50% in cases involving HD compared to 90.9% in cases with other diseases. Cases of cecal volvulus did not undergo nonoperative management.

Recurrence of volvulus occurred in 3 (27.2%) out of 11 cases that had initial successful nonoperative managemnt. One case had short segment HD, one case had functional constipation, and another one had neuronal intestinal dysplasia. The earliest recurrence was five months, while the last was 11 months after initial success.

Rectal biopsy was performed during the initial management of CV in 17 (81%) cases. Three cases did not perform rectal biopsy initially; 2 cases had their rectal biopsy done after anastomotic leak while the third case performed rectal biopsy after recurrence of symptoms. Rectal biopsy was not performed in one case as it was previously diagnosed by pathological examination to have short segment HD before the attack of CV.

Surgical intervention was definite management in all cases without recurrence of symptoms. It was the initial step in cases with cecal volvulus and in a case with perforated sigmoid volvulus. Surgery was performed on an elective basis for cases with sigmoid volvulus unless nonoperative management failed. Anastomotic leakage was a complication in 2 cases who were diagnosed later as short segment HD.

### Cases with CV associated with HD*: *Table 1

**Table 1:** Cases had CV associated with HD

#	Sex	Age at Presentation of CV	Presentation	HD	Initial Management			Interval to Definitive Repair	Definitive Procedure	Follow-up
					Nonoperative	Operative	Complications			
1	Male	4 Years	Noncomplicated Sigmoid Volvulus	Not known	Failed	Sigmoid Colostomy and Rectal Biopsy		3 Months	Duhamel	Osmotic Laxatives
2	Male	7 Years	Noncomplicated Sigmoid Volvulus	Not known	Failed	Sigmoid Colostomy and Rectal Biopsy		3 Months	Duhamel	Osmotic Laxatives
3	Female	8 Years	Noncomplicated Sigmoid Volvulus	Not known	Failed	Sigmoidectomy and Re-anastomosis	Anastomotic Leakage, Colostomy and Biopsies	3 Months	Abdominal Soave	Osmotic Laxatives
4	Male	8 Years	Noncomplicated Sigmoid Volvulus	Not known	Failed	Sigmoidectomy and Re-anastomosis	Anastomotic Leakage, Colostomy and Biopsies	3 Months	Abdominal Soave	Osmotic Laxatives
5	Male	9 Years	Noncomplicated Sigmoid Volvulus	Not known	Succeeded	None	Recurrence after 5 Months (Successful Nonoperative followed by Rectal Biopsy)	6.5 Months	Laparoscopic assisted Transanal Soave	Osmotic Laxatives and Enemas
6	Male	7 Years	Noncomplicated Sigmoid Volvulus	Not known	Succeeded (followed by Rectal Biopsy)	None		6 Weeks	Duhamel	Smooth
7	Male	7 Years	Perforated Sigmoid Volvulus	Not known	None	Sigmoid Colostomy and Multiple Biopsies		3 Months	Duhamel	Osmotic Laxatives
8	Female	11 Years	Noncomplicated Sigmoid Volvulus	Previously known (by Rectal Biopsy)	Succeeded	None		6 Weeks	Laparoscopic assisted Transanal Soave	Osmotic Laxatives and Enemas
9	Male	8 Days	Noncomplicated Cecal Volvulus	Not known	None	Cecostomy and Multiple Biopsies		6 Months	Abdominal Soave	Smooth

*CV*: Colonic volvulus; *HD*: Hirschsprung's Disease

#### Cases, not known to have HD, presented with noncomplicated sigmoid volvulus

Noncomplicated sigmoid volvulus was the first presentation in 6 cases; 5 males and a female. Their ages ranged from 4 to 9 years. They were presented with abdominal pain, abdominal distention and absolute constipation. Nausea and/or vomiting were present in 3 cases. Abdominal radiograph showed a combination of coffee bean sign and liver overlap sign in one case.

- Nonsurgical management was initially attempted in 2 cases, but it was unsuccessful. They were surgically managed by sigmoid colostomy and rectal biopsy, which was followed by Duhamel procedure 3 months later.
- The diagnosis of HD was overlooked in 3 cases. Nonoperative management was unsuccessful in 2 cases. Sigmoidectomy and primary anastomosis were implemented. Re-exploration was attempted in both cases due to anastomotic leakage when a colostomy was performed and multiple biopsies, including rectal biopsy through the anus, were collected. A diagnosis of short segment HD was settled. The third case was discharged on laxatives after successful nonoperative management and disimpaction. The condition recurred 5 months later and was successfully managed nonoperatively. The diagnosis of short segment HD was confirmed by rectal biopsy and contrast enema. Laparoscopic assisted transanal heart shaped anastomosis was performed 6 weeks later.
- Nonoperative management was successful in one case. The diagnosis of short segment HD was confirmed by rectal biopsy and contrast enema. Duhamel procedure was attempted 6 weeks later***.***

#### A case, not known to have HD, presented with complicated ‘perforated’ sigmoid volvulus

Perforated sigmoid volvulus was the first presentation in 7 years old male child. He was presented with toxic facies and a picture of peritonitis. Plain x-ray abdomen (erect view) showed free air under diaphragm. Exploration revealed perforated sigmoid volvulus. A sigmoid colostomy was carried out. Pathological examination of the multiple biopsies taken during exploration revealed short segment HD. Three months later, Duhamel procedure was successfully performed to him.

#### A case, previously diagnosed by rectal biopsy and barium enema to have short segment HD, presented with noncomplicated sigmoid volvulus

One case, 11 years female, was previously known to have short segment HD. She and her family refused surgery and continued rectal irrigations. She was presented with abdominal distention, pain, absolute constipation and vomiting. Plain abdomen x-ray showed coffee bean sign. Nonoperative management was successful. Laparoscopic assisted transanal heart shaped anastomosis was accomplished 6 weeks later***.***

#### A case, not known to have HD, presented with noncomplicated cecal volvulus

Cecal volvulus was the first presentation in a male neonate 8 days old. He was presented with refusal of feeding, abdominal distension and bilious vomiting. Abdominal x-ray showed dilated small bowel loops, a massively dilated loop in the right hypochondrium and absent distal aeration. Upon exploration, there was cecal volvulus and long cecal mesentery. A diagnosis of long segment HD up to the level of the transverse colon was made based on pathological findings. Cecostomy was performed to fix the cecum, followed by abdominal Soave at the age of 6 months.

## Discussion

CV is a rare presentation in the pediatric age group, especially during neonatal and infancy periods. A lot of cases can be misdiagnosed or mismanaged due to the rarity and the insidiousness of the disease. Moreover, its intermittent manifestations may delay the diagnosis. In the current study, 61.9% of cases reported previous similar attacks without asking for medical advice. Improper management can lead to colonic gangrene and life-threatening sepsis.

Along the gastrointestinal tract, the colon is the most affected part by volvulus. Although volvulus might affect the colon anywhere, sigmoid colon was reported as the most affected part of the colon [6]. Sigmoid colon was affected in 81% of our cases, while cecum was the part affected in 19% of cases. We did not report any case of transverse colon volvulus.

The etiopathogenesis of CV is unclear. There are several proposed predisposing factors, such as elongated mesentery with narrow attachment, long-standing constipation, HD, neurodevelopmental delay, anal stenosis and prune belly syndrome. Salas et al. suggested that long mesentery is the main risk factor [7]. Our findings suggested that redundancy and constipation are often intermingled in a vicious circle-like pattern. A loaded colon could progressively change in length and diameter, that is why redundant colon might be the long-term result of chronic constipation [8]. In contrast, the presence of a redundant colon would increase colonic transit time and slow emptying [9]. Noviello et al., found a significant increase in the rectosigmoid length in children with functional constipation [10]. It was not clear which came first, both redundancy and constipation might be a cause or a result.

HD, a common cause of constipation in neonates and infants, is considered a significant predisposing factor to CV, primarily through the progression of constipation. The incidence of HD is approximately 1:5000 live birth. About 90% of cases are diagnosed before the age of 5 years. In milder forms, however, diagnosis may be delayed until 10 years of age or even into adulthood [11]. Folaranmi et al. hypothesized that untreated constipation with the consequent loaded, heavy and dilated bowel will result in stretching and elongation of the fixing ligaments [12]. Mild cases of HD with delayed diagnosis often suffer from refractory constipation for many years before being diagnosed. The resultant progressive sigmoid dilatation in such cases may precipitate sigmoid volvulus.

The incidence of CV associated with HD varied among studies. The incidence in the current review (42.9%) was higher than that reported by Destro et al. (27.3%) [4]. Salas et al. reported HD in 17.5% of cases with sigmoid volvulus [7]. The relatively higher incidence of sigmoid volvulus associated with short segment HD in our series was attributed to the delay in diagnosis, with the median age at diagnosis being 7 years. In our opinion, delayed diagnosis—and consequently delayed management—was the main risk factor of CV in our cases with short segment HD. In contrast, congenital malfixation and a long mesentery were considered the main risk factors of CV in cases with long segment HD, which typically present earlier in life. In these cases, the affected colonic segment is usually proximal to the sigmoid colon, either the transverse colon or the cecum, depending on the level of aganglionosis and the malfixed segment.

HD as a predisposing factor should be considered in the differential diagnosis of every pediatric case presented with CV. In the current series, CV was the first presentation in 8 out of 9 cases had associated HD. Anastomotic leak occurred in 2 cases with sigmoid volvulus and missed diagnosis of short segment HD. A systematic review showed a significant increase in re-explorations when performing surgeries for CV without the suspicion of HD [13]. The role of rectal biopsy as a cornerstone in the management of cases with CV cannot be underestimated so as not to miss the diagnosis of short segment HD.

Prompt diagnosis of CV mandates a high index of suspicion, detailed history, meticulous examination and imaging studies. Marine et al. reported 44% diagnostic ability of plain abdominal radiograph; however, the presence of significant localized colonic dilatation with absent distal aeration would be helpful [14]. Contrast enema had a powerful diagnostic value through the appearance of the bird’s beak deformity which characterizes the cutoff of the twisted bowel. Nevertheless, contrast enema was not helpful in diagnosing HD during the acute attack. In our series, interval contrast enema was performed in patients with CV associated with HD, proved pathologically by rectal biopsy, who had undergone successful nonoperative management.

**Fig. 3:**
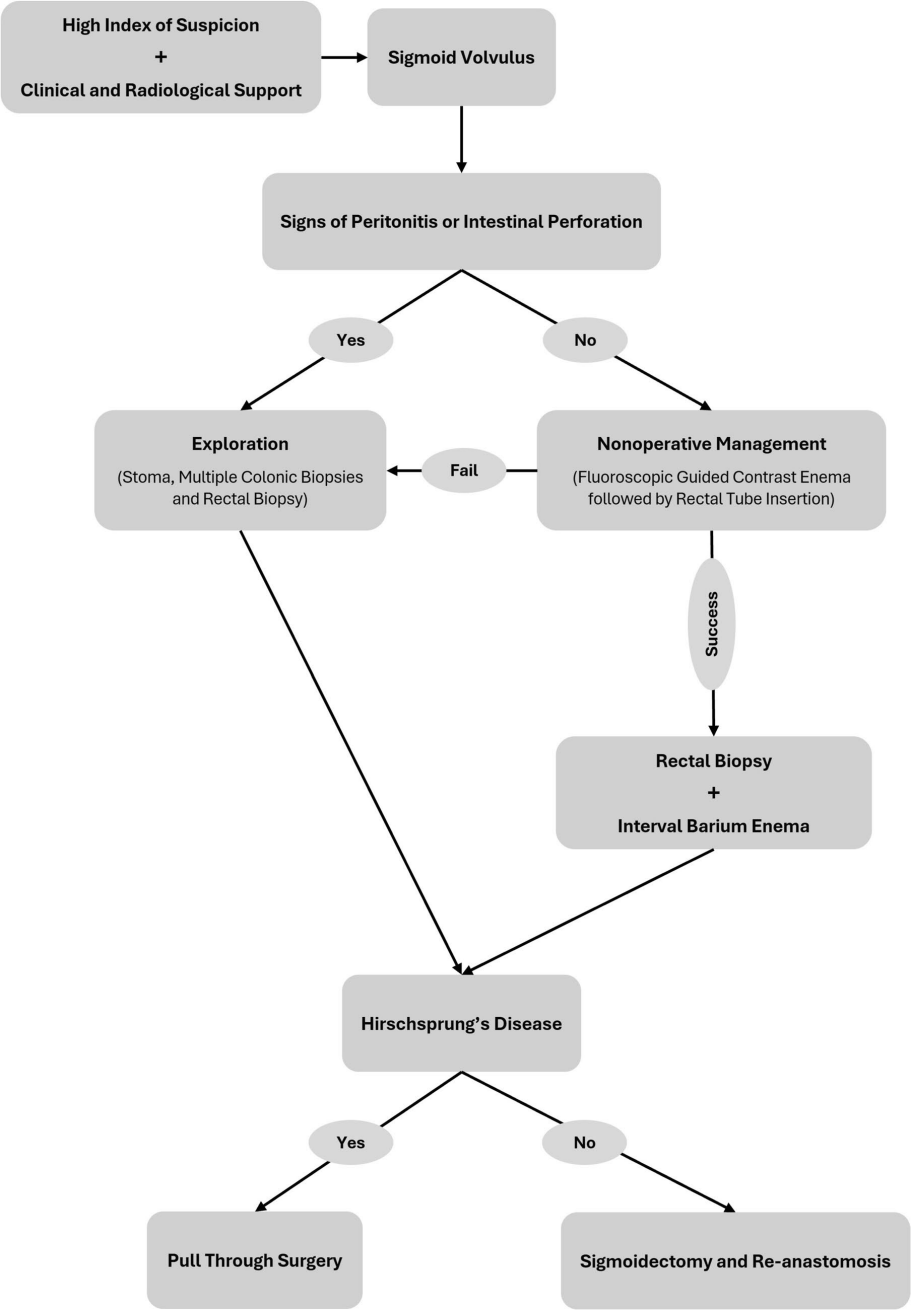
Proposed management algorithm for pediatric sigmoid volvulus

Contrast enema could successfully detort volvulus in early diagnosed, stable cases under fluoroscopic guidance if there is no peritonitis, bowel ischemia or perforation. The success rate was about 70–80% in the present and other studies [3, 7]. It was worth mentioning that the success of contrast enema to detort sigmoid volvulus was greatly affected in cases with HD. Furthermore, nonoperative management might carry a significant risk of recurrence [15]. Definite surgical repair of HD is the ultimate management of CV associated with HD. The role of nonoperative management should be limited to bypassing the acute attack. If failed, surgical exploration and colostomy would be preferred over resection and primary anastomosis. The rectal biopsy should be considered in all patients with CV before definitive treatment to rule out HD. A proposed management algorithm for pediatric sigmoid volvulus is illustrated in Fig. 3.

In our series, definitive surgical management of HD was attempted approximately 6 weeks after the initial management of CV in patients without peritonitis, whereas it was delayed up to 3 months in patients who either presented with peritonitis or developed it as a complication following unsuccessful initial management of CV. We observed that the occurrence of CV did not influence the choice of pull through technique, which was determined by the patient assessment and the preference of the operating surgeon. Furthermore, we noticed that these patients had a higher tendency toward postoperative constipation compared with HD cases without volvulus. This necessitated long-term use of osmotic laxatives, with rectal enemas used occasionally to facilitate evacuation.

The current study was limited by its retrospective design, small number of patients, differences in surgical management and its long period during which changes in care were possible. A multicenter, large database review is required to declare the true incidence and determine outcomes.

## Conclusion

The diagnosis of CV in children mandates a high index of suspicion. Definitive surgery should be done as soon as possible to avoid recurrence. HD is one of the most important predisposing factors to CV in children, and hence It should be suspected and excluded in every case. The accurate and prompt diagnosis of HD in cases with CV might alter the definite surgical procedure and hence decrease the postoperative complications.

## Data Availability

No datasets were generated or analysed during the current study.
